# A Bayesian Two Part Model Applied to Analyze Risk Factors of Adult Mortality with Application to Data from Namibia

**DOI:** 10.1371/journal.pone.0073500

**Published:** 2013-09-16

**Authors:** Lawrence N. Kazembe

**Affiliations:** Department of Statistics and Population Studies, University of Namibia, Windhoek, Namibia; National Taiwan University, Taiwan

## Abstract

Despite remarkable gains in life expectancy and declining mortality in the 21st century, in many places mostly in developing countries, adult mortality has increased in part due to HIV/AIDS or continued abject poverty levels. Moreover many factors including behavioural, socio-economic and demographic variables work simultaneously to impact on risk of mortality. Understanding risk factors of adult mortality is crucial towards designing appropriate public health interventions. In this paper we proposed a structured additive two-part random effects regression model for adult mortality data. Our proposal assumed two processes: (i) whether death occurred in the household (prevalence part), and (ii) number of reported deaths, if death did occur (severity part). The proposed model specification therefore consisted of two generalized linear mixed models (GLMM) with correlated random effects that permitted structured and unstructured spatial components at regional level. Specifically, the first part assumed a GLMM with a logistic link and the second part explored a count model following either a Poisson or negative binomial distribution. The model was used to analyse adult mortality data of 25,793 individuals from the 2006/2007 Namibian DHS data. Inference is based on the Bayesian framework with appropriate priors discussed.

## Introduction

The improvement of health of the population has been a long-term focus in most developing countries, mostly with the bid to meet the Millennium Development Goals [1]; [2]. African countries have channeled considerable resources aimed at boosting child and maternal health. National governments and development agencies have proposed strategies of advancing service delivery to effectively combat poor health in children and mothers through disease prevention and control [1]. Little focus, though, has been drawn on adult health, specifically on adult survival and mortality. In some countries adult mortality has slumped considerably, despite the remarkable gains in life expectancy and declining mortality in the 21st century [3]. The strong impact of HIV on mortality, and the survival of adults has to some extent substantially affected the population structure of African communities [2]; [4], Ngom. The life-course effects of abject poverty have created structural spheres which have negatively impacted on the quality of life manifesting at old age, further exacerbating adult mortality. Moreover, many factors including behavioural, socio-economic and demographic variables work simultaneously to impact on risk of mortality. Understanding risk factors of adult mortality is crucial towards designing appropriate public health interventions.

Notwithstanding, the paucity of vital statistics in most sub-Saharan Africa makes it difficult to study patterns and trends, as well as risk factors of adult mortality. In most sub-Saharan Africa, mortality data is based on census data, however, census are many years apart to inform meaningful trends and patterns, and assess the effect of any mitigatory interventions. To fill the gap, estimates or projections from the WHO/UNDP have been used [1]. In some countries, adult mortality estimates are derived from demographic surveillance system (DSS), where these exist INDEPTH or through the vital registration system (VRS). Ideally, the VRS is not functional in many African countries. An alternative to this is to use an increasing cross-sectional body of data publicly available from Demographic and Health Surveys (DHS), which collects data on survivorship/widowhood/siblings. Recently, the DHS has collected data in eight African countries, including Namibia, under the module “Support For Those Who Have Died” which captured the household census mortality data [6].

This study was aimed at understanding adult mortality by developing models that explain risk factors of adult mortality in Namibia. Following the conceptual framework as outlined in Rogers et al. [7]; [8], we incorporated a number of relevant explanatory variables, in particular, behavioural and health factors, socio-economic and demographic variables that shape mortality levels in a community. Within this framework, behavioural and health factors include alcohol drinking and smoking, availability and access to health care. The economic and social variables encompass level of living, marriage and family characteristics, and social ties to communities e.g. religion. Demographic variables consist of age, sex and ethnicity. These variables are further grouped into individual, household and community factors. Contextual factors, which measure unobserved or unmeasured determinants, are introduced as random effects. These are assumed to have a structured and unstructured pattern, where the structured effects permit similarities across areas, while the unstructured effects allow within area heterogeneity.

The need for geographical analysis is critical as it may assist in social planning by highlighting areas of excess mortality. To assume homogeneity in terms of regional mortality would be an understatement. The 2010 poverty estimates in Namibia, the country of focus in this study, indicated huge disparities across regions [9]; [10]. Moreover, the thirteen regions of Namibia remain disparate geographically, economically, culturally and socially and undoubtedly may explain the expected differentials in adult and old-age mortality (AMOA). Even so in these regions, there are considerable heterogeneity in health outcomes and socio-economic factors [10].

Past work on the epidemiology of adult mortality have considered either prevalence alone (i.e. whether death occurred in a household) and used logit models to analyze such [11]; [12], or severity only (i.e. compared the number of reported deaths) and applied count regression models [13]–[15]. In some cases where count data have excess zeros, zero-inflation (ZI) regression model have been used, particularly in general adult health [16]. Use of such ZI model in adult mortality is rare despite many examples in biostatistics, epidemiology and econometrics that deal with zero-inflated data. See for example Winkelmann [17] and references therein.

In this article, however, our proposal was that there are two processes: (i) whether death occurred in the household (prevalence part), and (ii) number of reported deaths, if death did occur (severity part). Wickelman [17] called such two-part processes as extensive (the zeros) and intensive (the positives) margins in a multi-index count model, representing the case that death did not occur and that death did occur, respectively. It is also referred to as a zero-hurdle model because it allows for a systematic difference in the statistical process governing individuals (observations) below the hurdle and individuals above the hurdle set at zero [17]–[19]. An alternative approach to two-part process is to use finite mixtures, which is a combination of zeros point mass distribution and the nonzero distribution [17]. Most work in this area, however, do not address concerns that occurrence of death (prevalence) and number of deaths (severity) reported in a household are joint processes, and that failing to account for the joint nature of these processes can bias estimates of risk factors on adult mortality [20]; [21]. Furthermore, several challenges arise in analyzing these data including how to: (i) handle correlation in the data between the prevalence and severity processes; (ii) model excess zero counts in the data; (iii) permit possible spatial correlation; and (iv) fit nonlinearity in metrical variables.

In this paper we proposed a structured additive two-part random effects model for adult mortality data to address the above issues. Therefore following Neelon et al. [18], Winkelmann [19], Olsen and Schafer [20] and Su et al. [21], the proposed model specification consisted of two generalized linear mixed models (GLMM) with correlated random effects, where the first part assumed a GLMM with a logistic link and the second part explored a count model following either a Poisson or negative binomial distribution. Furthermore, the random effects in the model permitted structured and unstructured spatial components to account for spatial correlation at area level. The proposed model was then used to analyse adult mortality data of 25,793 individuals from the 2006/2007 Namibian DHS data. Inference was based on the Bayesian framework with appropriate priors discussed.

The remainder of this paper is organized as follows. Section 2 presents the data, as well as outlines the two-part model and its application to the Namibian DHS data. Results of the analysis are given in Section 3. This is followed by the discussion in Section 4 and we conclude.

## Methods

### Data

This study used data from the 2006/2007 Namibia DHS [6], which included a household mortality module that has been implemented in 8 African countries since 2005 under the sub-theme of “Support for Persons Who Have Died”. The DHS are periodic cross-sectional health surveys funded by the U.S. Agency for International Development (USAID) Bureau for Global Health. The DHS have been conducted in over 70 countries since 1980s, and the data are publicly available at MEASURE DHS website. The data are completely anonymized before they are released for general public use. The DHS follows a cross-sectional multi-stage stratified survey design. In the Namibian context, a two-stage stratified sampling design was implemented to collect the data and provide direct estimates of demographic and health indicators at national and regional level. At first stage, a total of 500 enumeration areas (EA) from a sampling frame of 3750 EAs as defined in the 2001 Population and Housing Census, were selected stratified by urban-rural status with sampling probability proportional to the population of the region. At the second stage, a fixed number of 20 households were randomly drawn from each selected EA. From the selected households, all women of age 15–49 years were eligible for interview. A final representative probability sample of 10,000 households was selected. The sample allocation by region is given in the Appendix A of the Namibian DHS report [6]. The overall women response rates for urban and rural areas were 90.0% and 94.8% respectively, while for the 13 regions ranged from 88.4% (Khomas region) to 96.6% (Omusati region). The household response rate was 96.8% and 98.5% for urban and rural areas respectively, with a regional variation of 95.1% (Khomas region) to 99.1% (Oshikoto region).

The household module enumerated all usual household members and collected information on their age, gender and economic activities (education level, or employment status if not in school). All deaths in the household that occurred in the past 12 months preceding the survey were collected with full information on age at death and gender. The questions used to collect mortality data were: “Has any usual member of your household died in the last 12 months”, and if yes this was followed by “How many members died in the last 12 months”. Information were also collected on whether any household member was sick, whether any medical services, emotional or psychological, material or social support were available to the sick or deceased or any household member. In addition, approximate time to nearest health facility and type of health facility where health care was obtained were equally captured.

### Statistical Analysis

The number of household members that died was assumed to follow two process, which was better envisaged as a two-part mixture consisting of a point mass at zeros followed by truncated count data distribution for the non-zero observations. The model for the count can be either a Poisson or a negative binomial. These two-part models are also referred to as zero-hurdle models [18]; [19]. In particular, according to Winkelman [17], a hurdle model combines a dichotomous model for the binary outcome of the count being below or above the hurdle (the selection variable), with a truncated model for outcomes above the hurdle. For the Poisson hurdle model we have 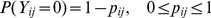
(1)

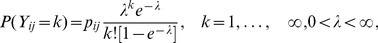
(2)where 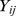 indicates the response for household member 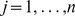 in area 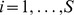 and 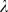 is the mean for the truncated Poisson distribution. An alternative to the Poisson hurdle is a negative binomial which replacing equation (2) is given by



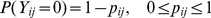
(3)

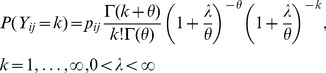
(4)with parameters 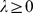 for the mean and 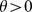 for over-dispersion. The component 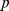 is a mortality propensity, while the count part models severity of mortality in the reported household. If one takes a less strict view of the two-part process then a finite mixture of two distributions can be assumed. A less strict view is assumed where zeros are generated from the same underlying process as positives, thus single-index models e.g. Poisson or negative binomial may apply. Otherwise, if the zero-generating process is not subject to such a constraint, then multi-index models are appropriate [17]; [19]. In this case the factors that explain whether death occured in a household within past 12 months may differ from the factors that determine the number of deaths following the first death in the same household.

A special case of the models above is the zero-inflated Poisson or zero-inflated Negative binomial. These models have a degenerate distribution at zero with untruncated Poisson or Negative binomial distribution. A zero-inflated Poisson is denoted by 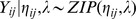. Combining zero inflation and over-dispersion gives a zero inflated negative binomial defined as 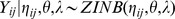, where 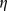 and 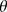 are the predictor and over-dispersion parameters respectively.

The zero-hurdle model can be extended to accommodate covariates and random effects. Since we have two parts, we introduced two GLMMs. For the prevalence component, we assumed a logit link while for the severity component we proposed a log link:
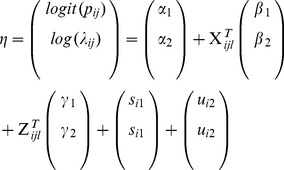
(5)where 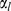 is the intercept for process 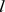, the terms 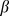 = (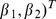 are vectors of regression parameters corresponding to the set of covariates, 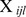 (Table 1). The non-linear components of continuous covariates (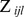) were captured through terms 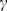 = (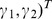. The components 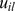 and 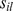 are the unstructured heterogeneity and spatially structured variation terms, respectively, at regional level. Because of dependence in the binary and count outcomes, the random effects were correlated and were modelled using multivariate distributions as explained below.

**Table 1:** Summary of household members who died by selected characteristics.

Variable	Percentage died	Total	χ^2^ test (p-value)
*Residence*			
Urban	5.4	10829	157.7 (<0.001)
Rural	9.7	14964	
*^1^Nearest health facility*			
Hospital	6.3	5531	54.5 (<0.001)
Health centre	8.6	1880	
Clinic	8.5	17903	
*^2^Means to nearest health facilit*			
Car/motorcycle	4.8	4013	75.6 (<0.001)
Public transport/Animal cart	8.0	4877	
Walking	8.7	16021	
*^3^ime to nearest health facility*			
Minutes	6.6	16730	135.9 (<0.001)
Hours	10.6	8174	
Day	12.1	612	
*Sex of household head*			
Male	6.0	15019	16.6 (<0.002)
Female	10.6	10774	
*Sex of household member*			
Male	7.3	12020	12.8 (<0.001)
Female	8.5	13773	
*^4^ducation of household member*			
None	8.5	3876	115.4 (<0.001)
Primary	9.9	8230	
Secondary and higher	6.5	13283	
*^5^Marital status of household member*			
Married	5.4	9464	203.7 (<0.0001)
Never married	8.8	13796	
Others	13.4	2162	
*Wealth quintile*			
Poorest	12.0	4215	299.2 (<0.0001)
Poor	9.8	4785	
Medium	8.8	6126	
Rich	6.5	6134	
Richest	2.9	4533	

Numbers and percentage missing per variable are provided at the foot of the table.

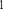
Missing: 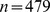 (1.9%).

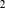
Missing: 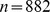 (3.1%).

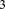
Missing: 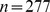 (1.1%).

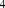
Missing: 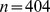 (1.7%).

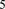
Missing: 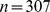 (1.3%).

Model estimation was carried out using the Bayesian approach and the following prior distributions were specified for all parameters of the model (5). For the intercept, diffuse priors were assumed, that is, 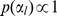, while for the other fixed effects, 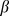, highly dispersed normal distribution priors were chosen, that is, 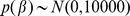. The smooth functions of continuous covariates were modelled using a second-order random walk prior given by 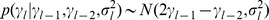 for 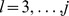 with noninformative priors for the initials. Again 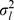 controlled the amount of smoothing, with larger values leading to less smoothing. The unstructured spatial effects 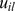 were assumed to follow a multivariate normal distribution, i.e., 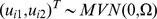, with covariance matrix 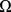. The spatial structured effects 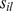 were assigned a multivariate conditional autoregressive (MCAR) prior, i.e., 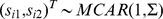, again 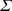 is a covariance matrix [18]; [22].

The covariance matrices have their diagonal elements equal to the variances and the off-diagonals are correlation components between the outcome processes. Thus, for example 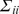 is variance components, while 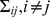 are cross-covariance components between the prevalence of mortality (part 1) and severity of mortality (part 2). Correspondingly, 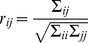, for example, gives a measure of spatial correlation between the processes. The variance components were assigned inverse Wishart priors, i.e., 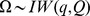, 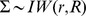 where 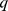, 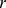 are scalars, while 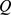, 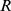 are symmetric and positive definitive matrices. The hyperpriors were assigned 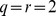, 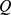 = 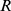 = 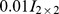 where 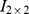 is an identity matrix.

The regression tool for full Bayesian inference was based on the posterior distribution of all parameters. Markov Chain Monte Carlo techniques were used to draw samples from the full conditionals of all parameter distribution which were then summarized to obtain model estimates in the posterior analysis, i.e., 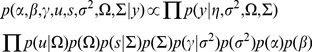
(6)where 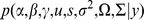 is the joint distribution of all parameters in the observation model and the corresponding priors, and 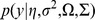 is the likelihood for all observable data (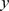). Gibbs sampler was used to draw samples from the full conditionals.

Model implementation was carried out in WinBUGS 1.4 [23]. The sample code used for our bivariate problem was adapted from Neelon et al. [18]. See Appendix S1. We fitted a Poisson and negative binomial hurdle models. For each model, three separate chains were run to help assess convergence, starting from different initial values for the priors. As pointed out by Gelman [24], the performance of the model is sensitive to the choice of the hyperparameters. We therefore considered alternative specifications on variance components hyperparameters, 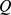 and 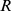, and carried out sensitivity of our model, by assuming 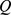 = 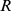 = 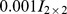, 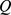 = 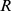 = 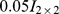, and 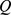 = 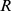 = 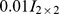. The results were similar, therefore, the last choice was maintained. Convergence was monitored by visual examination of time series plots of samples for each chain, and confirmed by plotting the Gelman-Rubin statistic. The first 5,000–10,000 samples were discarded as a “burn-in” and then each chain was run for a further 30,000 iterations or till convergence was achieved. Models were compared using the Deviance Information Criterion (DIC: Spiegelhalter et al. [25]), which was simultaneously computed in the estimation process. Smaller values of DIC indicated a better fitting model.

## Results

**Figure 1:**
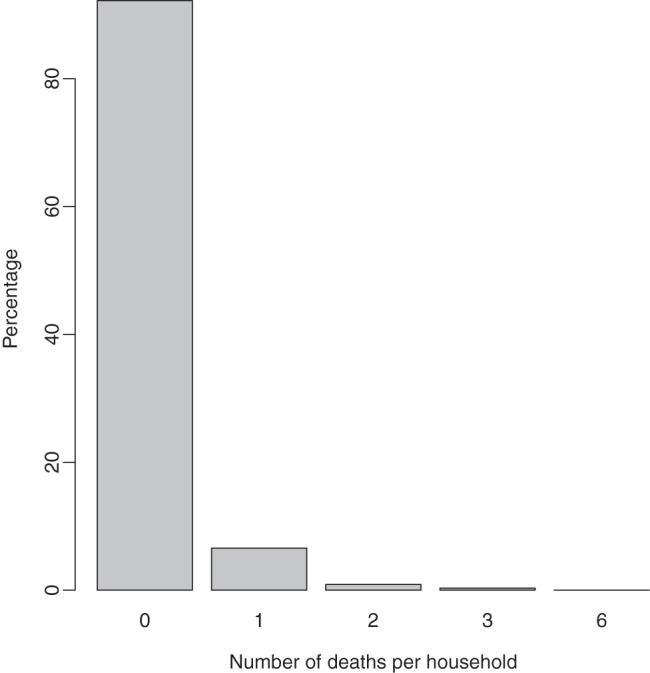
Histogram of percentage of reported adult and old-age mortality in Namibia, 2006/07 DHS.

**Figure 2:**
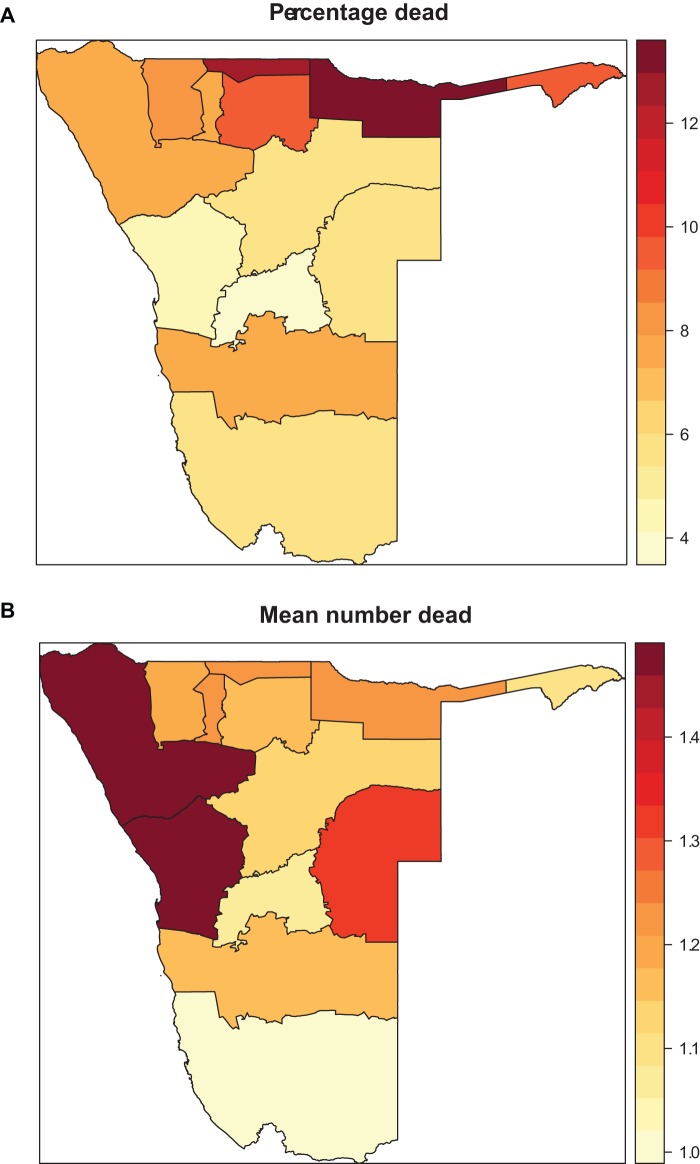
(a) Percentage of household with at least one member dead within 12 **months of the survey date by region; (b) Mean number of household members reported dead by region.**

Figure 1 displays the age-specific histogram of adult and old-age deaths. As proposed the number of reported zero deaths was high consisting of 92% of the total observations, with 1 death given in about 6.6% household, while 2, 3 and 6 deaths reported per household represented 0.8%, 0.3% and 0.01% respectively. Figure 2 shows the percentage who died by region (panel (a)) and the mean number of persons reported dead per household by region (panel (b)). Table1 presents a descriptive summary of percentage who died by socio-demographic covariates. Relatively small numbers were missing for some variables mainly due to item non-response. There was significant difference across categories. In part, there were more deaths in rural households, in female-headed households and in the poorest of the poor households, and the nearer the health facility the fewer the deaths reported.

We fitted four models, two were of random effects only based on the Poisson and Negative binomial, and the other two extended the two random effects models by including fixed and nonlinear effects. The DIC was used to for model selection. The DIC values are given in Table 2. The basic Poisson logit hurdle had a 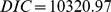 (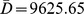, 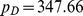) which was of poor fit compared to the basic NB logit hurdle (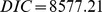). Comparatively, models with fixed and nonlinear effects outperformed the basic models. However, the extended NB logit hurdle model was superior to the extended Poisson logit hurdle (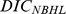  = 8439.92 versus 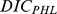  = 9739.95 respectively).

**Table 2:** Model comparison values based on Deviance Information Criterion (DIC) for the Poisson logit hurdle and negative binomial (NB) logit hurdle models.

Model	Description	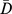	*p_D_*	*DIC*
Poisson logit hurdle	Region (Random effects: RE)+ Cluster (RE)	9625.65	347.66	10320.97
Poisson logit hurdle	Fixed + Nonlinear + Region (RE) + Cluster(RE)	9008.61	365.67	9739.95
NB logit Hurdle	Region (RE) + Cluster(RE)	7799.10	339.06	8577.21
NB logit Hurdle	Fixed + Nonlinear+ Region (RE) + Cluster(RE)	7741.38	349.27	8439.92

Table 3 presents posterior summaries from the Negative binomial hurdle model. Given are the fixed and random effects for both the logit and negative binomial components. Odds of death in the family was positively associated with female headed households (log odds of 0.48, 95% CI: 0.38, 0.59), negatively with being married (−0.48, 95% CI: −0.64, −0.32) and never married (−0.21, 95% CI: −0.38, −0.04), positively with being poorest (0.95, 95% CI: 0.60, 1.28), poor (0.81, 95% CI: 0.46, 1.11), medium (0.98, 95% CI: 0.67, 1.26) and rich (0.78, 95% CI: 0.54, 1.04) relative to being the richest, increased when one takes hours to reach a hospital (0.65, 95% CI: 0.60, 1.28), and positively if the family head had primary education (0.18, 95% CI: 0.06, 0.23).

**Table 3:** Posterior means (post. mean) for Fixed and Random effects estimates with corresponding 95% credible intervals (CI) from the spatial negative binomial hurdle model of adult mortality.

Variable	Bernoulli		Negative binomial	
	Post. Mean	Post. 95% CI	Post. Mean	Post. 95% CI
*Fixed effects*				
Constant	−4.73	(−5.44, −4.22)	−4.45	(−5.02, −3.91)
Urban	−0.23	(−0.54, 0.08)	−0.35	(−0.66, −0.07)
Rural	0		0	
Hospital	0.21	(−0.06, 0.61)	0.09	(−0.20, 0.41)
Clinic	0.29	(−0.01, 0.61)	0.15	(−0.11, 0.42)
Health centre	0		0	
Walk	0.05	(−0.14, 0.31)	0.16	(−0.05, 0.35)
Public transport	0.17	(−0.06, 0.38)	0.23	(0.02, 0.43)
Car/motorcycle	0		0	
Female head	0.48	(0.38, 0.59)	0.33	(0.23, 0.42)
Male head	0		0	
Female member	0.005	(−0.08, 0.16)	0.02	(−0.07, 0.09)
Male member	0		0	
Married	−0.48	(−0.64, −0.32)	−0.37	(−0.54, −0.21)
Not married	−0.21	(−0.38, −0.04)	−0.14	(−0.30, 0.02)
Other (married)	0		0	
Poorest	0.95	(0.60, 1.28)	0.75	(0.47, 1.09)
Poor	0.81	(0.46, 1.11)	0.75	(0.47, 1.05)
Medium	0.98	(0.67, 1.26)	0.83	(0.58, 1.12)
Rich	0.78	(0.54, 1.04)	0.73	(0.51, 0.99)
Richest	0		0	
Time to facility (Min)	0.33	(−0.17, 0.90)	0.29	(−0.17, 0.79)
Time to facility (Hr)	0.65	(0.14, 1.27)	0.62	(0.18, 1.12)
Time to facility (Day)	0		0	
No education	−0.001	(−0.19, 0.16)	−0.03	(−0.19, 0.08)
Primary education	0.18	(0.06, 0.31)	0.12	(0.01, 0.23)
Secondary and higher education	0		0	
*Random effects*				
Spatial structured				
(Σ_11_,Σ_22_)	0.13	(0.002, 0.58)	0.44	(0.06, 1.58)
(Σ_12_)	0.88	(0.59, 1.31)	-	
Spatial unstructured				
(Ω_11_, Ω_22_)	1.69	(1.25, 2.04)	1.77	(1.40, 2.20)
(Ω_33_)	0.34	(0.21, 0.59)	-	

With regards to severity of mortality at household level, we observed that risk of deaths decreased in urban areas (−0.35, 95% CI: −0.66, −0.07), increased if public transport was used (0.23, 95% CI: 0.02, 0.43), increased in female headed households (0.33, 95% CI: 0.23, 0.42), was higher in all socio-economic strata other than in the richest stratum, was positively associated with primary education (0.12, 95% CI: 0.01, 0.23), and increase if time to facility was in hours (0.62, 95% CI: 0.18, 1.12).

The variance components for the random effects, in Table 3, showed strong spatial correlation in the prevalence estimated as 0.13 (95% CI: 0.002, 0.58), while severity correlation was 0.44 (95% CI: 0.06, 1.58). The covariance was estimated as 0.88 (95% CI: 0.59, 1.31) showing a strong correlation between prevalence and severity. For the unstructured variance component we obtained 1.69 (95% CI: 1.25, 2.04) and 1.77 (95% CI: 1.40, 2.20) for the logit and NB parts respectively. The unstructured covariance for the two components (prevalence and severity) was 0.34 (95% CI: 0.21, 0.59). The calculated correlation coefficients for the structured and unstructured components were 0.04 and 0.09 respectively.

**Figure 3:**
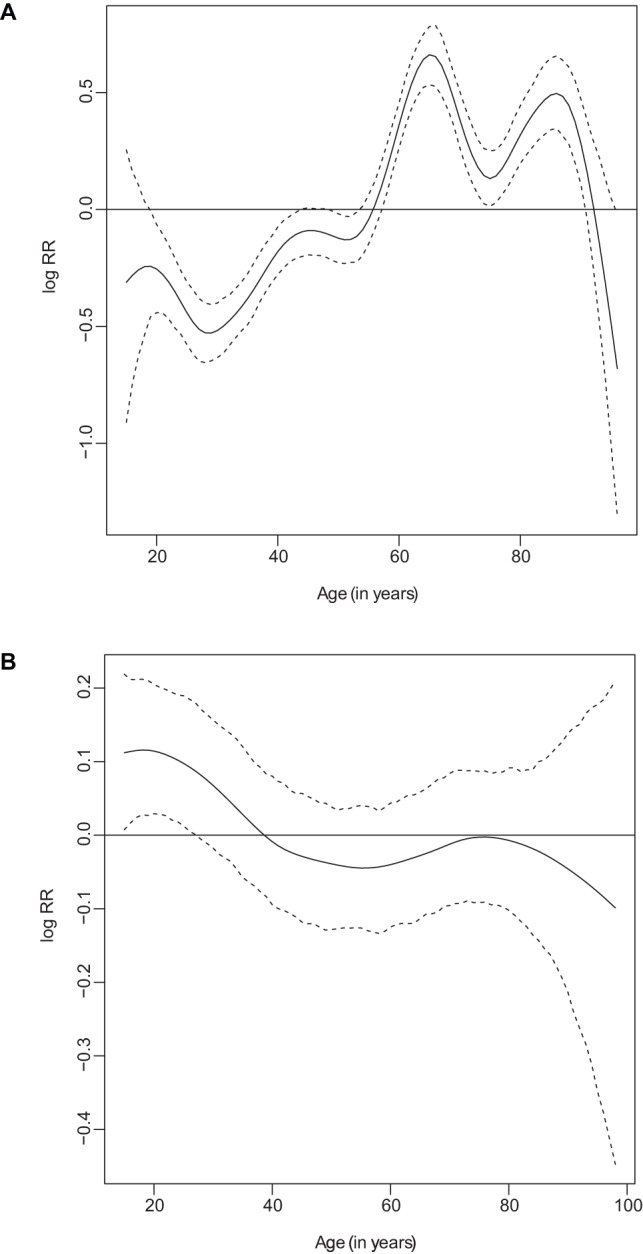
Nonlinear effects of: (a) age of household head; and (b) age of household member, given by the solid centre line as log relative risk (RR) with corresponding 80% confidence band (dotted outer lines).

Figure 3 displays age curves for the household head and household member for the count component [since the logit and count components yield similar curves]. In both panels we observed a significant departure from zero, as well as from linearity, although this was more pronounced in age of household head (Figure 3a). In the left panel, the risk of mortality increased between 15–20 years and then decreased up to age 30 years, then rose again steadily up to age 65 years, with a little dip at age 50 years. A similar pattern of up and down continued from age of 65–70 years with a final decrease at age of 80 years. From age of 15 years to 55 years, the risk of death lied below zero, suggesting a reduced mortality risk in such households, while at 60 years to the end we observed a risk of above 0, indicating an increased risk of mortality. Overall the dip in risk was at age of 30 years, and a peak in risk was at 65 years. For the age of household member (Figure 3b), there was an overall decreasing risk with increasing age. Up to age 30–35 years the risk was significantly above zero, an age of increased mortality among household members. From age of 40 years, although the risk remained below zero, this remained non-significant based on the confidence bands. Overall a drop in risk was noted at age of 50 years, while a peak in mortality was displayed at age 80 years.

**Figure 4:**
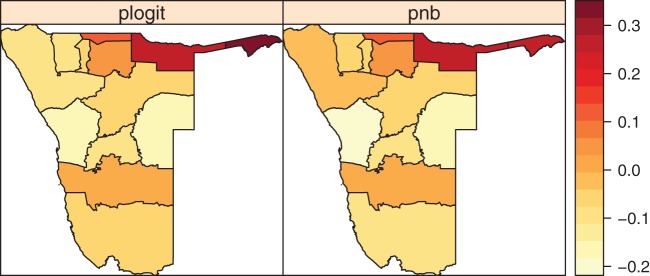
Regional model estimates of residual total spatial effects for the logit part (left panel) and the negative binomial count part (right panel).

**Figure 5:**
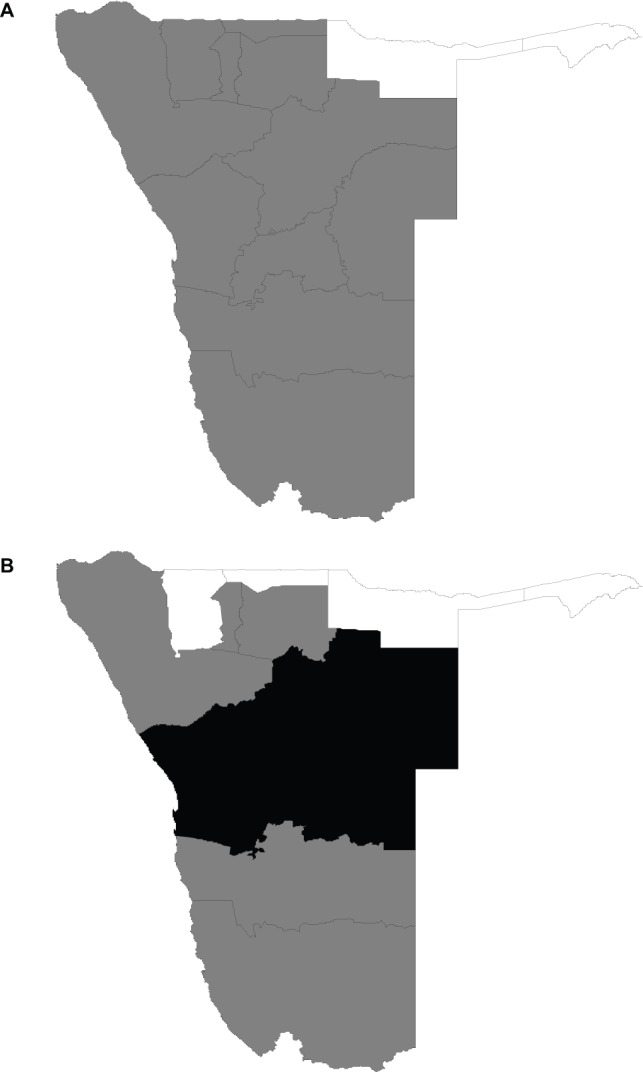
Significance effects of region for a nominal level of 80%. (a) Posterior probabilities for logit part; (b) Posterior probabilities for NB part. Black denotes regions with strictly negative credible intervals. White denotes regions with strictly positive credible intervals. Grey denotes regions with no significance association with the outcome.

Figure 4 presents residual spatial effects for both model components. As the patterns from the two plots suggest there were similarity in risk between the logit (prevalence) model and negative binomial (severity) model. The increased odds or risk of mortality were observed in the Caprivi and Kavango regions, while decreased odds and risk of mortality were predicted in Erongo and Omaheke regions. The other regions clearly showed a predicted risk not different from zero i.e 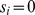. Figure 5 displays the significance map corresponding to the spatial effects given in Figure 4. The significance map displays three colour schemes: black, white and grey. Black colour denotes regions with strictly negative credible intervals, whereas white denotes regions with strictly positive credible intervals and grey denotes regions with no significance association with the outcome. For the logit spatial effects (Figure 5a), only two regions, Caprivi and Kavango, had significant positive effects implying that the odds of adult mortality were significantly higher in these regions than in others. For the NB part (Figure 5b), we obtained both positive and negative significance areas. Positively significant effects were obtained in Caprivi, Kavango, Ohangwena and Omusati regions suggesting that severity of mortality was higher in these regions compared to others. Regions with negatively significant effects were in Erongo, Khomas, Otjozondjupa and Omaheke. This means the risk of adult mortality was relatively lower in these four regions than in others.

## Discussion

We presented a bivariate or two-part model estimating risk factors of adult and old-age mortality in Namibia based on a nationally representative health survey, 2006/07 DHS, which captured mortality and survivorship data of all members in sampled households. This is a rich source of data, which produced consistent mortality estimates when compared to the census or indirect estimates of WHO and UNDP, despite limitations due to sampling errors and selection bias. We refer interested readers to the work by Bendavid et al. [3] which presented tables showing comparison of various estimates using WHO, UNDP and this source.

Our approach used the two-part model by assuming two processes governing mortality observed at households. We conjectured that the process of death occurring (extent) would be different from those influencing multiple deaths (intensity) in the households reporting deaths. We therefore postulated that the risk set, although similar, will have different association for the two processes. Indeed, the different significance covariates obtained in the two sets shows that this is the case. For example “urban” and “public transport” were not significant on the logit model, yet these were significant on the count model (Table 3). Similarly, variable like “not married” was significant on the logit model but was not on the count model. The significance of “urban” under the severity model indicates that that although death may occur at a particular household, the risk of repeat within a year is significantly lower in urban areas than in rural areas. Moreover, the difference in magnitude of the estimates between the logit and count model (Table 3), suggests that the odds of death occurring is relatively higher than the risk of observing multiple deaths in same household.

In estimating risk factors of adult mortality, we have included both individual and household variables as demand-side factors, and clinic factors as supply-side factors within the conceptual framework of health care [26]. That is, here we assumed that adult mortality is aggravated by availability and access to health care [27]; [28]. However, the epidemiology of adult mortality is not this simplistic. It is an interaction of a myriad of factors, be it health care, socio-economic, demographic and behavioural factors. Despite limited variables in the DHS we have included variables that fall within all these risk categories. In fact, we extended our model to include spatial random effects, purporting that unobserved or unmeasured covariates present other potential source of risk to mortality. The significance of spatial effects does support our proposition that disparities in health are engrossed in Namibian regions [9]; [10]. These results should generate further research to unearth potential risk factors of varied adult health in the country.

From a statistical point of view, several issues arise here. First, we used a bivariate conditional autoregressive process with permits correlation between the logit and count model. Without such an approach, a two-part model would give biased estimates [18]; [21]. Second, we applied a structured additive regression (STAR) model in an attempt to explain the complex relationship between adult mortality risk and various risk factors. The use of such models is increasingly being applied in epidemiology. See Kazembe [10]; Neelon et al. [18]; Fahrmeir and Lang [29] and references therein. STAR models simultaneously model spatially structured random effects, unstructured random effects, nonlinear effects of metrical covariates, together with the usual fixed categorical variables. Third, with respect to the likelihood, the zero-augmented models show a better fit to the data, moreover, the observed zero counts are well captured in Hurdle and ZINB as discussed in Zeileis et al. [30].

Our analysis, however, has the following limitation. While the use of structured and unstructured spatial effects provides robust spatial estimates when the locations are many, in this analysis, we only have 13 provinces to estimate spatial effects and most of the provinces do not have more than one neighbour. This may bias the spatial pattern observed here. Moreover, the large regional areas may conceal or over-step variability of risk within that region in which all areas within are depicted as having common risk of mortality. An ideal analysis would be to use small-areas (districts or constituencies) to assess spatial variability in adult mortality, unfortunately these spatial units were not available at the time of this analysis, but would be worthwhile to pursue this further.

Nevertheless, the results of this study has various important implications. First, it might assist epidemiologists with understanding potential risk factors of adult mortality that needs to be factored in when planning interventions. Second, it could help understand the health consequences of social inequality, human behaviour and demographic factors on adult mortality, which in turn is crucial towards comprehending population dynamics [8]. Third, it might help social planners with understanding where resources should be targeted. Fourth, it might generate hypotheses as to factors explaining spatial variability in adult mortality, be it HIV epidemic which has a strong age-specific impact on adult mortality [2]; [4].

In conclusion, this paper explored the use of advanced structural additive models to study adult mortality risks. Our study used the most recent data, although six years old, notwithstanding provide a glimpse of multivariate relationship existing between various factors. It provides a natural picture of forces affecting adult mortality, in contrast to the UNDP/WHO indirect estimates. There is need to consider the risk of cause-specific adult mortality. For example, it may be of interest to ascertain what is the spatial distribution of HIV related mortality as this is the major cause of adult mortality in Namibia [2]. However, the lack of HIV related factors in data in general, and in the model in particular, is possibly missing a lot of information and makes it difficult to assess the significance of such factors which is crucial toward informing further public health effort. Be as it may, multilevel models can be used to incorporate reliable HIV related data often aggregated at areal level. Furthermore, multivariate models that categorize death into communicable, non-communicable and accidents/injuries will be worthwhile investigating to explicitly display the spatial variability of risk of adult mortality by cause.

## Supporting Information

Appendix S1 **WinBugs Code.**
(DOCX)
