# Supplementary material for: A Bayesian Two Part Model Applied to Analyze Risk Factors of Adult Mortality with Application to Data from Namibia
Source: PLoS One. 2013 Sep 16;8(9):e73500. doi: 10.1371/journal.pone.0073500 (PMC3774685; doi:10.1371/journal.pone.0073500)
Supplement: Appendix S1 — WinBugs Code. (DOCX) [file pone.0073500.s001.docx]

**Appendix S1**

WinBUGS code for fitting a Bayesian two-part model for adult mortality. Adapted from Neelon et al. [18].

model {

K <-10000 # Constant for implementing zeros trick

for (i in 1:N) {

## Likelihood ##

p[i]<- max(0.001,min(0.999,q[i]))

logit(q[i])<- beta1[1]+ beta1[2]*urban[i]+beta1[3]*hospital[i]+beta1[4]*clinic[i]+ beta1[5]*walk[i]+beta1[6]*pubtrans[i]+alpha1[1]*fhead[i]+alpha1[2]*fmember[i]+gamma1[1]*age1[i]+gamma1[2]*age2[i]+gamma1[3]*age3[i]+gamma1[4]*time1[i]+gamma1[5]*time2[i]+gamma1[6]*time3[i]+Phi[1,region[i]]

## Note: age1-time3 are spline basis functions imported from R ##

log(mu[i])<- beta1[1]+ beta1[2]*urban[i]+beta1[3]*hospital[i]+beta1[4]*clinic[i]+beta1[5]*walk[i] +beta1[6]*pubtrans[i]+alpha1[1]*fhead[i]+alpha1[2]*fmember[i]+gamma1[1]*age1[i] +gamma1[2]*age2[i]+gamma1[3]*age3[i]+gamma1[4]*time1[i]+gamma1[5]*time2[i]

+gamma1[6]*time3[i]+Phi[2,region[i]]

#Log-likelihood

z[i]<-step(y[i]1) ## I(y>0)

ll[i]<-(1-z[i])*log(1-p[i]) + z[i]*(log(p[i]) + y[i]*log(mu[i]) - mu[i]- loggam(y[i]+1) - log(1-exp(-mu[i])))

zeros[i]<-0

zeros[i] ~dpois(phi[i]) ## Zeros trick

phi[i] <- ll[i]+K

}

## Priors ##

beta1[1]~ dflat() ## Intercepts

beta2[1]~dflat()

for (j in 2:5) {

beta1[j] ~dnorm(0,.1) ## Patient-level fixed-effect parameters

beta2[j] ~dnorm(0,.1)

}

for (j in 1:2) {

alpha1[j] ~dnorm(0,.1) ## Block-level fixed-effect parameters

alpha2[j] ~dnorm(0,.1)

}

for (j in 1:6) {

gamma1[j] ~ dnorm(0,.1) ## B-Spline coefficients

gamma2[j] ~ dnorm(0,.1)

}

## Bivariate CAR Prior for Phi

Phi[1:2,1:n] ~ mv.car(adj[],weights[],m[],R[,]) ## m specifies no. of neighbors

for(i in 1:M)

{

weights[i]<-1

} ## M is the sum of the vector m

## Spatial Precision and Covariance

R[1:2, 1:2]~dwish(Omega[,], 2) ## Omega = diag(2) and included as part of data

Sigma.phi[1:2,1:2]<-inverse(R[,])

rho<-Sigma.phi[1,2]/sqrt(Sigma.phi[1,1]*Sigma.phi[2,2])

}
